# Pancreatic ductal adenocarcinoma and distal cholangiocarcinoma: a proposal of preoperative diagnostic score for differential diagnosis

**DOI:** 10.1186/s12957-021-02120-w

**Published:** 2021-01-12

**Authors:** Edoardo Maria Muttillo, Antonio Ciardi, Raffaele Troiano, Paolina Saullo, Gabriele Masselli, Marianna Guida, Alessandra Tortora, Isabella Sperduti, Giulio Marinello, Piero Chirletti, Roberto Caronna

**Affiliations:** 1 Department of Surgical Sciences, Sapienza University of Rome, Viale del Policlinico 155, 00161 Rome, Italy; 2 Department of Radiological Sciences, Oncology and Pathology, Sapienza University of Rome, Italy, Viale del Policlinico 155, 00161 Rome, Italy; 3 Biostatistical Unit – Clinical Trials Center, IRCCS Regina Elena National Cancer Institute, Via Elio Chianesi 53, 00144 Rome, Italy

**Keywords:** Differential diagnosis, Primary pancreatic adenocarcinoma, Primary distal cholangiocarcinoma, Preoperative diagnosis, Pancreatic head carcinoma, Pancreaticoduodenectomy

## Abstract

**Purpose:**

The differential diagnosis between primary adenocarcinoma of the pancreas head and distal cholangiocarcinoma remains a clinical challenge. Recent studies have shown important differences in terms of survival between these tumors. Therefore, different treatments should be considered, but the preoperative histological diagnosis is still difficult. Aim of this study is to create a preoperative diagnostic score for differential diagnosis between primary pancreatic adenocarcinoma and primary distal cholangiocarcinoma.

**Methods:**

One hundred eighty consecutive patients who underwent pancreaticoduodenectomy at Sapienza University of Rome from January 2010 to December 2019 were retrospectively analyzed. Inclusion criteria were pancreatic or biliary histologic origin obtained by definitive postoperative histological examination. Exclusion criteria were diagnosis of ampullary carcinoma, non-ampullary duodenal adenocarcinoma, pancreatic metastasis, and benign disease. One hundred one patients were considered eligible for the retrospective study. Preoperative biological, clinical, and radiological parameters were considered.

**Results:**

CRP > 10 mg/dL (*p* = 0.001), modified Glasgow Prognostic Score 2 (*p* = 0.002), albumin < 35 g/L (*p* = 0.05), CA 19-9 > 230 U/mL (*p* = 0.001), and Wirsung diameter > 3 mm (*p* < 0.001) were significant at univariate logistic analysis. Multivariate logistic analysis has shown that parameters independently associated with primary pancreatic adenocarcinoma were CRP > 10 mg/dL (*p* = 0.012), CA 19-9 > 230 U/mL (*p* = 0.043), and diameter of the Wirsung > 3 mm (*p* = 0.005). Through these parameters, a diagnostic score has been developed to predict a primary pancreatic adenocarcinoma when > 1 and a primary distal cholangiocarcinoma when < 1.

**Conclusion:**

This feasible and low-cost diagnostic score could have a potential impact to differentiate pancreatic cancer histologic origin and to improve target therapeutic strategy.

## Introduction

Distal cholangiocarcinoma (DC) and pancreatic ductal adenocarcinoma (PDAC) are two different pancreatic head malignancies in close anatomic proximity. Although they share similar therapeutic strategies and the same surgical resection (pancreaticoduodenectomy, PD), DC and PDAC have shown different long-term oncologic outcomes [1, 2]. The clinical differential diagnosis is still a challenge because they share many symptoms and the same radiologic patterns but specific biomarkers are not available [3].

Currently, many procedures are used to obtain histological preoperative diagnosis (percutaneous ultrasound-guided core needle biopsies, ERCP with biliary brushing, and endoscopic ultrasound-guided (EUS) biopsy). The European Society of Gastrointestinal Endoscopy (ESGE) recommends performing EUS-guided sampling as a first-line procedure when a pathological diagnosis is required. Potential advantages of EUS-FNA compared to other procedures consist of facilitated immunostaining and better capability to diagnose specific tumor types and lower risk of seeding (3% vs 16%). However, these procedures are characterized by a high rate of failure for the detection of malignancy (14%), and their use for histological differential diagnosis is limited by the suitability of the sample [4–7]. For these reasons, in the guidelines N.C.C.N 2019, a preoperative biopsy is not recommended [8].

However, it seems contradictory to propose the same therapeutic strategy for two different cancers with different clinical evolution and prognosis.

Given the real difficulty in obtaining a preoperative differential diagnosis (PDAC/DC), this study aims to assess the diagnostic value of these parameters and obtain a preoperative diagnostic score. This paper analyzed many clinical, biological, and radiological parameters, yet present in literature, that are usually subject of study for pancreatic head neoplasm in order to evaluate preoperative patients’ operability, neoplasm’s resectability, and risk of postoperative complications [9–14].

## Methods

### Subject selection and areas of study

For this retrospective study, consecutive 180 patients who underwent PD at Sapienza University of Rome from January 2010 to December 2019 were analyzed. The inclusion criteria were pancreatic or biliary histologic origin obtained by definitive postoperative histological examination. Out of them, 79 cases with a diagnosis of ampullary carcinoma, non-ampullary duodenal adenocarcinoma, pancreatic metastasis, and benign disease were excluded. In total, 101 patients, divided into 66 PDAC and 35 DC, were enrolled. Preoperative parameters were divided into three groups: clinical, biological, and radiological. The *clinical parameters* included gender, age, ASA score, and BMI (BMI > 25 as cut-off value were considered). The *biological parameters* commonly revealed preoperatively (analyzed within 1 month before the operation) were CA 19-9 value, nutritional and inflammatory markers such as albumin, CRP, modified Glasgow Prognostic Score, neutrophil-lymphocyte ratio (NLR), and platelet-lymphocyte ratio (PLR). Jaundice patients with high bilirubin level (> 10 mg/dL) and high value of CA 19-9 were excluded to prevent influence on marker validation. *Radiological parameters* included Wirsung duct diameter measured at greatest dilatation point, pancreatic density, and pancreatic attenuation index (PAI) as a ratio of pancreatic and splenic density. As reported, a Wirsung duct > 3 mm was considered dilatated [9]. Mean density values of the pancreas and spleen were calculated in basic conditions. The density value, differently to Yardmici et al. which calculate the density in six different points of the pancreatic body and tail, has been calculated automatically by system positioning on a region of interest (ROI) where the parenchyma was most represented, making every effort to avoid the pancreatic duct and extrapancreatic structures. Cut-off value ≥ 40 HU was considered as high density and < 40 HU as low density and an average ratio pancreas/spleen (PAI) respectively < 0.54 as low and 0.54 as high [9, 10].

### Statistical analysis

Descriptive statistics was used to summarize pertinent study information. Associations between categorical variables were analyzed according to the Pearson chi-square test or Fisher exact test, when appropriate. The odds ratio (OR) and the 95% confidence intervals (95% CI) were estimated using the logistic regression univariate model. A multivariate logistic regression were developed using stepwise regression (forward selection, enter limit and remove limit, *p* = 0.10 and *p* = 0.15, respectively), to identify independent predictors of outcomes. The assessment of interactions between significant investigational variables was taken into account when developing the multivariate model. Significance was defined at the *p* value less than 0.05 level.

The log-OR obtained from the multivariate model were used to derive weighting factors of a continuous prognostic index, aimed to identify differential outcomes’ risks. Coefficient estimates were “normalized” dividing by the smallest one and rounding the resulting ratios to the nearest integer value [15]. Thus, a continuous score assigning to patients an “individualized” risk was generated. The score was dichotomized according to prognosis with the ROC analysis (the best “splitter” cut-off is determined) [16].

To address the multivariate model overfit and to validate the results, a cross-validation technique, which evaluates the replication stability of the final multivariate model in predicting all outcomes, was also investigated, using a resampling procedure [17].

This technique generates a number of simulation datasets (at least 100, each approximately 80% of the original size), by randomly selecting patients from the original sample, to establish the consistency of the model across less-powered patient’ samples. Risk classes were generated on the basis of the combination of the found risk factors.

The ROC analysis allowed to assess the predictive accuracy of the prognostic model, by the AUC determination [18]. The SPSS (version 21.0; SPSS, Inc., Chicago, IL) and MedCalc (version 14.2.1; MedCalc software, Ostend, Belgium) licensed statistical programs were used for all analyses.

## Results

The study group (101 patients) was composed of 62 males and 39  females with a mean age of 69  years (range 44–87). There were 38 patients with BMI > 25 and 63 patients with BMI ≤ 25. A prevalence of ASA score 2 and patients with resectable tumors [19] were observed as reported in Table 1. For each parameter, we have considered cut-off values according to the literature [20–22]. The results of univariate logistic regression odds ratio models for predictors of PDAC or DC are shown in Table 2. Univariate analysis identified 5 parameters as diagnostic for primary pancreatic adenocarcinoma (PDAC), including modified Glasgow Prognostic Score > 1, CRP > 10 mg/dL, Wirsung duct > 3 mm, CA 19-9 > 230 U/mL, and albuminemia < 35 g/L.

**Table 1:** General features of patients

Parameters	Category	Number	Percentage
Gender	Male	62	61.4
	Female	39	38.6
Age	Mean (range)	69	(44–87)
BMI	> 25	38	37.6
	< 25	63	62.4
Histologic origin	Pancreas (PDAC)	66	65.3
	Distal cholangiocarcinoma (DC)	35	34.7
Resectable/BR	Resectable	85	84.16
	Borderline resectable	16	15.84
ASA	I	8	7.9
	II	59	58.4
	III	33	32.7
	IV	1	1

Data are expressed as *n* (%) unless otherwise specified

*ASA* American Society of Anesthesiologists, *BMI* body mass index

**Table 2:** Univariate and multivariate analysis

Preoperative parameters	Mean ± SD	Category	Number (%)	Univariate	OR	Multivariate	*p*
				***p value***		***95% CI***	
**Clinical**							
Age	69 (8.87)	≤ 69	33 (32.7)	0.52			
		> 69	62 (61.3)				
ASA	2.26 (0.6)	≤ 2	67 (66.3)	0.78			
		> 2	34 (33.7)				
Gender		Male	62 (61.4)	0.45			
		Female	39 (38.6)				
BMI		≤ 25	38 (37.6)	0.63			
		> 25	63 (62.4)				
**Biological**							
Albuminemia (g/L)		< 35	50 (49.5)	0.05			n.s.
		≥ 35	51 (50.5)				
**CRP (mg/dL)**		**≤ 10**	**54 (53.5)**	**0.001**	**3.65**	**(1.32–10.11%)**	**0.012**
		**> 10**	**47 (46.5)**				
mGPS		0–1	66 (65.3)	0.002			n.s.
		2	35 (34.7)				
NLR		≤ 2.7	43 (42.5)	0.64			
		> 2.7	58 (57.5)				
PLR		≤ 146	43 (42.5)	0.42			
		> 146	58 (57.5)				
**CA 19-9 (U/mL)**		**≤ 230**	**49 (48.5)**	**0.001**	**2.752**	**(1.03–7.33%)**	**0.043**
		**> 230**	**52 (51.5)**				
**Radiological**							
**Wirsung diameter (mm)**		**≤ 3**	**34 (33.6)**	**< 0.0001**	**4.068**	**(1.54–10.7%)**	**0.005**
		**> 3**	**67 (66.4)**				
HU		≤ 40	78 (77.2)	0.13			
		> 40	23 (22.8)				
PAI		< 0.54	35 (34.6)	0.35			
		≥ 0.54	66 (65.4)				

After multivariate analysis, only three factors remained as independent predictors of PDAC: CA 19-9 > 230 U/mL, CRP > 10 mg/dL, and Wirsung duct > 3 mm (Table 2).

**Fig. 1:**
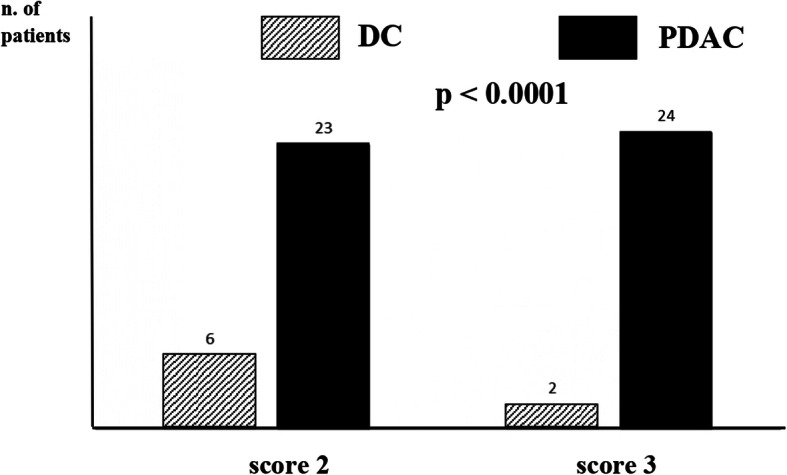
PDAC diagnosis was achieved in 80% of the cases with a score of 2 and in 96% of the cases with a score of 3

A preoperative diagnostic score was then developed. The score ranges from a minimum 0 to a maximum of 3 points with cut-off estimated at 1 through the ROC curve. When the score is > 1, a diagnosis of PDAC can be predicted, while a diagnosis of DC can be predicted when the score is < 1 (Tables 3 and 4) with high accuracy (AUC 74%). PDAC diagnosis was achieved in 80% of the cases with a score of 2 and in 96% of the cases with a score of 3, as shown in Fig. 1.

**Table 3:** Diagnostic score

Preoperative diagnostic score			
		Beta	Score
CRP	> 10 vs ≤ 10	1,297	1
Wirsung diameter (mm)	> 3 vs ≤ 3	1,403	1
CA 19-9	≤ 230 vs > 230	1,012	1

**Table 4:** Diagnostic score

Score	Diagnosis	OR	95% CI	AUC (SE)	*p* value
≤ 1	Distal cholangiocarcinoma	8.35	3.22–21.63	0.74 (0.05)	< 0.0001
> 1	Pancreatic adenocarcinoma				

*OR* odds ratio, *95% CI* 95% confidence interval, *AUC* area under the curve, *SE* standard error

## Discussion

Pancreatic-biliary cancers are extremely aggressive diseases with an increasing incidence worldwide [23]. The rate of survival after surgical resection remains poor. Only 15–20% of patients with PDAC have a resectable tumor because most of them are locally advanced and/or metastatic at the time of diagnosis [24]. Therefore, the rate of surgical resection is low because of late diagnosis. Then, due to their close anatomic proximity and similar management with pancreaticoduodenectomy, DC and PDAC are often treated as 1 entity. Currently, it is not recommended to perform a preoperative biopsy in *resectable tumors* because a differential diagnosis would not change the therapeutic strategy (upfront surgery) and could expose to the risk of disease progression [25]. However, for *borderline resectable tumors*, an EUS-guided biopsy is recommended in order to perform a target neoadjuvant chemotherapy. Given the histological similarity between PDAC and DC and the difficulties in obtaining suitable samples during EUS, definitive preoperative histological diagnosis is not often achieved and this may affect the outcome of surgical or radio/chemotherapeutic treatment. Although a detailed understanding of biologic behavior differences is lacking, recent studies have shown wide differences in terms of survival after surgical resection between PDAC and DC in terms of anatomopathological findings (N+, perineural invasion, grading, etc) and chemotherapy responsiveness [26–28]. Therefore, the diagnostic and therapeutic paths of PDAC and DC are becoming progressively different. In fact, in PDAC, the use of neoadjuvant chemotherapy is increasingly considered even in resectable tumors [29, 30], while in DC, primary surgical resection remains the gold standard for resectable tumors [31].

Given the importance of preoperative knowledge of histologic origin, this study has been proposed to correlate some preoperative parameters with postoperative histological diagnosis.

In univariate analyses, PDAC were associated with modified Glasgow Prognostic Score > 1, CRP > 10 mg/dL, Wirsung duct > 3 mm, CA 19-9 > 230 U/mL, and albuminemia < 35 g/L.

At multivariate analysis, the parameters independently associated with the PDAC were Wirsung duct > 3 mm (*p* = 0.005), CA 19-9 > 230 U/mL (*p* = 0.043), and CRP > 10 mg/dL (*p* = 0.012), and from these parameters, the diagnostic score has been developed.

These parameters, commonly evaluated in terms of prognosis, in this study were correlated for the first time with histological diagnosis. The correlation between pancreatic histologic origin and high value of CA 19-9, mostly related to risk of recurrence and to locally advanced tumors, reflects the PDAC malignant potential. It has been also reported as a predictive marker of tumor staging/resectability, and furthermore, several reports have suggested that CA 19-9 serial measurement can predict chemotherapy response [32–34].

About Wirsung duct dilatation, often studied as a protective factor for pancreatic fistula, this paper disclosed a correlation with PDAC and could reflect an early duct involvement in PDAC compared to DC, due to the different primary origin [35]. This aspect could also explain the high rate of exocrine pancreatic insufficiency observed in PDAC and the substantial contribution of malnutrition in determining outcomes on PDAC patients [36].

The association between high CRP to PDAC remains innovative: in fact, this inflammatory marker has been associated with poor survival after resection and with low resectability rate but never with histologic origin (primary pancreatic adenocarcinoma, PDAC) [37, 38].

In the previously exposed scenario, the score proposed in this study could be useful, especially when the differential diagnosis is not even achieved through radiological and/or EUS-guided findings and in patients with 2 or 3 score value.

The benefit of knowing the histological origin may be significant if one considers that this information can modify the therapeutic approach and timing. In fact, in primary pancreatic cancer (PDAC), neoadjuvant chemotherapy is achieving widespread acceptance in borderline and resectable patients, taking also a role in selecting patients who may or may not be candidates for surgery [29, 30]. However, the same strategy seems not suitable in distal cholangiocarcinoma (DC): applying “untarget” neoadjuvant chemotherapic protocols could expose DC patients to disease progression (unresponsive patients) and make them unresectable [31]. Considering that we often do not know preoperatively the histologic origin of pancreatic head cancers, any effort to distinguish PDAC and DC could be done to plan which is the best therapeutic approach (upfront surgery/neoadjuvant chemotherapy) and to select the best preoperative chemotherapic protocol, if indicated, without confusing PDAC and DC.

Finally, this score presents some advantages: easily obtainable because composed by routinely radiological and biological parameters, easily feasible (not require any technological equipment), no risks of complications, no risk of delaying treatment, and no additional cost.

There are however some limits: this is a retrospective study and analyzes a limited sample of patients, although highly selected.

## Conclusion

Discriminating PDAC from DC is mandatory in order to set the correct therapeutic strategy and to avoid non-target treatments. However, there are some problems related to diagnostic procedures to obtain a preoperative differential diagnosis. Therefore, the use of this diagnostic score is an original proposal and could be useful to select the best treatment. Although this score has a high level of accuracy, it will certainly have to be validated through prospective studies and could be also implemented with other data (EUS features, biomolecular markers).

## Data Availability

The datasets used and/or analyzed during the current study are available from the corresponding author on reasonable request.

## Abbreviations

DC: Distal cholangiocarcinoma
PDAC: Pancreatic ductal adenocarcinoma
PD: Pancreaticoduodenectomy
ERCP: Endoscopic retrograde cholangio-pancreatography
EUS: Endoscopic ultrasound
ESGE: European Society of Gastrointestinal Endoscopy
EUS-FNA: Endoscopic ultrasound fine needle aspiration
ASA: American Society of Anesthesiologist
BMI: Body mass index
CA 19-9: Cancer antigen 19-9
CRP: C-reactive protein
NLR: Neutrophil to lymphocyte ratio
PLR: Platelet to lymphocyte ratio
PAI: Pancreatic attenuation index
ROI: Region of interest
HU: Hounsfield unit
OR: Odds ratio
CI: Confidence intervals
AUC: Area under the curve
ROC: Receiver operating characteristics
N.C.C.N: National Comprehensive Cancer Network
